# Glasgow Royal Infirmary

**Published:** 1829-10-01

**Authors:** 


### XXXIV.

### GLASGOW ROYAL INFIRMARY.

### I. DISEASE OF THE MAXILLARY SINUS.*

We have often expressed much pleasure at the zeal with which our northern confrères attached to public institutions have of late endeavoured to make their experience available to others by clinical reports. Dr. Ballingall, of Edinburgh, and the surgeons of the Glasgow Infirmary, really deserve much credit for the perseverance they have shewn in running this good race, which must lead to honest satisfaction on their own parts, and hearty approbation from their brethren. But dropping these pleasing considerations for the present, we proceed to advert to the *matériel* of a report of the surgical cases treated in the Glasgow Infirmary, from the 1st of November, 1828, to the 1st of May, 1829, by Dr. Couper, senior surgeon to the Establishment.

Inflammation of the lining membrane of the Highmorian antrum, says Dr. Couper, sometimes terminates, not in suppuration, but in copious deposition of thick mucous matter, filling up the whole of the cavity. Its speedy removal is essential to prevent ulceration of the mucous membrane, caries of the bony parietes, or fungus in the interior. The following case is advanced as the representative of the foregoing opinions.

*Case.* D. M'Queen, æt. 70, admitted Nov. 13th, 1828, with the right nostril occupied by a fungous tumour completely filling its cavity, and pushing the septum narium towards the left side. The fungus resembled, in appearance the common soft polypus of the nose, and bled when touched—right cheek tender and somewhat fuller than

---

* Glasgow Journal, No. VII. Aug.

the left—right side of the roof of the mouth softer and more prominent than the left—gums spongy, and most of the *teeth* (he was 70) *loose*, with slight purulent discharge oozing out from their sockets—right lachrymal sac much distended with constant flow of tears—right eye protruding about half an inch, with eversion of the lower lid, and chemosis of the conjunctiva, large and sluggish pupil, and somewhat impaired vision—hearing of right ear affected—occasional severe pain, commencing round the orbit, and extending over the whole right side of the scalp, most frequent at night, and accompanied with throbbing of the temples. These latter attacks were generally followed by bleeding at the nose, which afforded relief; the general health was good.

Twelve months previously he had received a blow above the right ear with a stick, which stunned him, and occasioned slight epistaxis. After this he became subject to head-ache, and in the course of three months, he noticed slight obstruction in the right nostril, succeeded by occasional hæmorrhages. Six weeks before admission, the vision of the right eye began to fail, but the eye-ball did not sensibly protrude till three months afterwards, since which period the symptoms, to use an American phrase, "progressed."

"The third and fourth molares on the diseased side being extracted, a perforation was made through their sockets into the antrum. Only a little blood issued; on introducing a probe no firm tumour was perceptible in the antrum, but the cavity was found to be filled with a soft pulpy substance, through which the probe could be passed freely in all directions, coming into contact with a small extent of bare bone in the direction of the orbit. On injecting tepid water through the perforation, it issued freely by the nostril, bringing away, besides blood, a quantity of a white substance, of the consistence of butter. The polypus being removed from the nostril with the forceps, tepid water was repeatedly injected through the perforation, until it issued unmixed from the nostril, and the quantity of inspissated mucus thus extracted, was greater than would have completely filled the antrum in its natural state. The injection with tepid water was afterwards continued daily, and occasional anodynes were given to alleviate the pain in the head. At the end of two weeks, the swelling of the cheek and palate, the protrusion of the eye-ball, the epiphora and ectropion, had disappeared; the vision of the right eye was completely restored, but he still heard imperfectly with the right ear, and suffered occasional attacks of pain in the right side of the head. At the end of two months, he left the hospital in perfect health, excepting occasional slight headache. There was then no discharge from the antrum, although the perforation had been carefully kept open, and no bare bone could be perceived with the probe."

### II. AFFECTIONS OF THE EYE.

### 1. *Case of Gonorrhœal Ophthalmia.*

J. Watt, æt. 26, admitted Dec. 10th, 1828, with the lids of both eyes greatly swollen and of purple colour; the conjunctivæ of dark red colour, much distended, and discharging a large quantity of thick yellow matter; state of corneæ not to be ascertained on account of the intolerance of light; most severe burning pain in both eye-balls, extending round the orbits, and greatly aggravated at nights, or when exposed to the light; pulse 108, of good strength; skin hot; tongue white. Four weeks previously he contracted gonorrhœa, for which he used some internal remedies, that at the end of a fortnight greatly diminished the discharge. Twelve days before admission, felt slight itching in both eyes, accompanied with heat and acrid discharge, and after two days the eye-lids became swollen and the discharge purulent. No change in the gonorrhœa took place when the eyes became affected, but even on admission he had some discharge from the urethra, unaccompanied with heat or pain.

"Although the first stage of the disease was past, it was thought requisite to apply leeches several times around both eyes, during the first two days after this patient's admission, and the application was followed by much relief of the pain. The eyes were frequently washed out with a syringe and tepid water, emollient and anodyne cataplasms were applied, vinum opii was dropped in the eyes, and blisters were placed on the temples and the nape of the neck, and kept open during the course of the disease. No stimulants were applied to the urethra, as the discharge from it continued unchanged. As the violence of the disease abated, which it did very slowly, the injections of tepid water were exchanged for a weak solution of corrosive sublimate in rose water, with the addition of a little vinum opii, and afterwards for a solution of lapis divinus; wine, tonics, and nourishing diet were also given, but it was not till two months from the time of admission that he was able to leave the hospital. He had then been about three weeks free from pain and discharge from the eyes. The left eye-ball was much shrunk in size, and its vision was entirely lost from exfoliation of the cornea. The right cornea had also suffered much from the same cause, but, a small portion of it still remaining lucid, he could see with this eye so as to number large objects held up before him, or guide himself through the ward. The discharge from the urethra continued through the whole course of the disease; and at the time of his dismissal was still so considerable that he required the use of an astringent injection for its removal.

"It may be fairly presumed, that the ophthalmia in this case did not arise from the direct application of gonorrhœal matter to the eyes, because the disease appeared exactly at the same time in both eyes, and the chances of simultaneous inoculation are not very considerable. It is also plain that the occurrence of the affection of the eyes was not an example of metastasis, because the gonorrhœa suffered no change on the appearance of the new disease, but ran its usual course, continuing even when the ophthalmia had ceased. It must be concluded, then, that this severe affection arose from a sympathy existing betwixt the eyes and the urethra, a sympathy, which fortunately operates only on rare occasions."

We do not deem it by any means conclusive that the disease was not produced by contagion, *because* both eyes were affected at the same time, but let that pass. The case is an instructive one, and shews well the ravages of this severe ophthalmia. The abominable carelessness and dirtiness of some patients is really astonishing; we lately saw a case of gonorrhœal inflammation of the eye, produced by the patient's having applied to it the same rag that he used for the penis!

2. *Cancer—Extirpation of the Eye-ball.*

James Bennie, æt. 50, admitted Feb. 9th, 1829, with a tumour the size of a kidney-bean, of cartilaginous hardness, and adhering pretty firmly to the bony margin of the orbit, at the outer canthus of the right eye. In its centre was an irregular ulcerated opening, discharging a quantity of curdy pus, but no denuded bone could be felt with a probe—it was the seat of lancinating pain, which frequently shot upwards along the forehead—the integuments around had a dull livid hue—the eyelids could only be separated to a small extent, and merely disclosed a white, opaque, rough surface, where the eye should be—general health good, with the exception of acidity of stomach. The disease commenced five years previously in the form of a pustule at the outer angle of the eye, which broke and formed a small irritable sore. The latter slowly extended and encroached on the eyeball, which gradually shrank, and the sight of which had been destroyed for twelve months before his admission.

The tumour, eyeball, and diseased portion of eyelids were extirpated with the hook and scalpel, the optic nerve only being cut with a pair of curved scissors. No bleeding took place, but the pain was excruciating, and was only appeased by a large anodyne. Suppuration took place in four days, the patient was dismissed with the wound cicatrized at the end of a month, and he now continues in perfect health.

We have to regret that no account of the dissection of the extirpated tumour is reported, and consequently that no opportunity is afforded the profession of judging whether the disease were really scirrhus. We need scarcely say that such accompaniments to cases of this kind are absolutely essential for the formation of a proper judgment on the issue. It is notorious that for want of such details many and many a fallacious cure of cancer has been recorded and believed, and patients actually affected with that disease, subjected on the strength of those histories to unnecessary, perhaps injurious, measures.

### III. Lithotomy after the Operation for Fistula Ani — Consequences of the Operation.

James Hardie, æt. 37, admitted March 11th, 1829, with frequent desire to make water, which was passed with much pain along the course of the urethra and neck of the bladder, especially when the last drops were voided—urine sometimes bloody, especially after exercise or the introduction of instruments into the bladder, but without any mucous or sandy deposit. A full-sized sound passed readily into the bladder, where it struck on a hard smooth substance, which was always found lying towards the right side, and could not be felt by the finger passed into the rectum; the prostate gland was natural. Extending from the anus forwards, and towards the left side, occupying part of the incision made in the lateral operation of lithotomy, was the cicatrix of an operation for fistula in ano, performed some years previously. The symptoms of stone commenced about sixteen years before his admission, but had never been urgent, till within the preceding fortnight, when they followed, as he imagined, the use of carbonate of soda internally.

"A few days after admission, the lateral operation was performed by running a straight probe-pointed bistoury along a curved staff, grooved on its convex side. A little more than the usual time was spent in cutting into the urethra, in consequence of the hardness of the cicatrix just mentioned, but, the stone being extracted on the first introduction of the forceps, the whole operation was finished within six minutes. The calculus weighed about an ounce and a half, and was composed of the phosphates of ammonia and magnesia. During the first few days no bad symptoms occurred. About the fifth day the wound began to look foul, and on the eighth day some flatus and a little feculent matter was observed to escape from it. On examination with the finger it was found that the old cicatrix had ulcerated, producing a small aperture betwixt the wound and the rectum, immediately above the sphincter. A bistoury was passed through the opening, and carried downward, so as to divide the sphincter. An elastic catheter was kept constantly in the urethra, and stimulant dressings were applied to the wound. Healthy granulations soon made their appearance, and at the end of seven weeks the cicatrization was complete, but a small opening into the urethra still remained, through which the greater part of the urine was discharged. On his return to the hospital, as directed, at the end of three months after the operation, the opening into the urethra had contracted so much, that no urine escaped by it, if he kept his thighs together during micturition, and even when he did not observe this precaution, the escape was trifling. It was proposed to apply the actual cautery for the purpose of closing the fistulous opening, but the inconvenience he suffered was so trifling that he declined to submit."

### IV. Fractures and Amputation.

Case 1.—*Fracture of the Radius, Olecranon, and great Trochanter.*

Clementina M'Gregor, æt. 29, of delicate habit, admitted with much swelling of the left wrist—fracture of the radius about three-fourths of an inch from its carpal extremity—transverse fracture of the olecranon, which was drawn considerably upwards—the soft parts round the elbow tense and greatly swollen, and the integuments over the fracture of a livid hue. Besides these various ills there were, swelling of the left buttock and upper third of the thigh—the limb about half an inch shorter than its fellow, and the toes everted—crepitus in the situation of the great trochanter on making extension or rotating the limb—acute pain in the hip on pressure or motion. The accident had happened five hours previously from a violent fall on her left side, in consequence of her foot having slipt in the street.

Inflammation being subdued by the usual means, splints and bandages were applied to the arm and thigh, both of which were placed in the straight position. The radius and femur united in seven weeks, but the olecranon required three months for its consolidation, and then much stiffness of the joint remained, which was diminished by friction and passive motion.

CASE 2. *Fracture of the Coronoid Process of the Ulna—Symptoms observed.* F. Ferguson, æt. 17, was attacked on the night of the 5th of April, and knocked down upon his left side, by which he severely injured the left elbow. On admission, two days afterwards, the following is the list of symptoms enumerated.

"At present there is much swelling and tension around the elbow joint. The olecranon is not fractured, but has been thrown upwards and backwards, and a considerable hollow is felt immediately above it. The upper head of the radius is dislocated outwards and backwards. The inner condyle remains attached to the shaft of the humerus, but the distance betwixt this condyle and the olecranon is increased about an inch. The external condyle cannot be felt from the situation of the head of the radius. The distance betwixt the olecranon and the acromion measures an inch less on the injured, than on the opposite arm. The forearm is kept bent at an obtuse angle with the humerus, and is in a state of pronation. He can, however, supinate the arm, although with difficulty and pain. By fixing the humerus, and making extension of the forearm, the joint can be made to assume its natural appearance, but the instant the extension ceases, the joint resumes the appearance just described.

"From these various particulars, which were ascertained gradually, as the swelling of the soft parts abated, it was concluded, that the coronoid process of the ulna was fractured, an injury of very rare occurrence. The fore-arm was placed across the chest, with the fingers pointing towards the opposite shoulder, and secured in this position with splints and a bandage. At the end of four weeks union had taken place, and was attended with little deformity, although some stiffness of the joint. The latter was diminishing, under the use of passive motion, when he left the hospital."

We are "free to confess," in the oratorical phrase, that the symptoms are not conclusive, at least to our minds, in determining the existence of a fracture of the coronoid process. The impossibility of feeling the external condyle is a somewhat suspicious item, and we rather suspect, that the injury was rather an oblique fracture of the lower extremity of the humerus, than what it is considered in the report. In cases like these, where very rare accidents are suspected, we think that unless the diagnosis is clear, the leaning should always be the other way; we mean that, cæteris paribus, the symptoms should be rather referred to the common than the extraordinary injury. The contrary practice introduces a dangerous laxity in diagnosis.

CASE 2. *Amputation at the Wrist-Joint for Compound Fracture of the Hand.* In a case of compound fracture of the hand from machinery, the fore-arm being unhurt, Dr. Couper resolved to amputate at the wrist-*joint*. This being fully extended, an incision was made with a narrow catline through the integuments, over and in the direction of the radio-carpal joint, when the wrist was bent, the instrument passed into the joint, and brought out in a direction obliquely downwards, so as to form a flap out of the upper part of the palm. The flap adhered well, and in three weeks "the stump was whole."

"This operation," says Dr. Couper, "appears to possess several advantages over the usual mode of amputating at the fore-arm. The motions of pronation and supination are better preserved, which is of consequence in the event of using an artificial hand. And lastly, the ends of the bones are better defended from injury, being covered with cartilage, nor does this circumstance, as far as appears from the present case, impede the healing of the wound." They must take the fact, who may not like the reasoning, in the above instance. The opinion that the ends of the bones being covered with cartilage is a protection, differs from the sentiment usually expressed by surgical authorities, and acted on in practice. However, there are more things in Heaven and Earth than are dreamt of in our philosophy, and now-a-days one learns to be surprised at nothing.

---

